# Development of Adenovirus Containing Liposomes Produced by Extrusion vs. Homogenization: A Comparison for Scale-Up Purposes

**DOI:** 10.3390/bioengineering9110620

**Published:** 2022-10-27

**Authors:** Jaimin R. Shah, Tao Dong, Abraham T. Phung, Tony Reid, Christopher Larson, Ana B. Sanchez, Bryan Oronsky, Sarah L. Blair, Omonigho Aisagbonhi, William C. Trogler, Andrew C. Kummel

**Affiliations:** 1 Moores Cancer Center, University of California San Diego, La Jolla, CA 92037, USA; 2 Department of Chemistry and Biochemistry, University of California San Diego, La Jolla, CA 92093, USA; 3 Materials Science and Engineering, University of California San Diego, La Jolla, CA 92093, USA; 4 Department of NanoEngineering, University of California San Diego, La Jolla, CA 92093, USA; 5 EpicentRx, Inc., La Jolla, CA 92037, USA; 6 Department of Surgery, University of California San Diego, La Jolla, CA 92037, USA; 7 Department of Pathology, University of California San Diego, La Jolla, CA 92037, USA

**Keywords:** adenovirus, cancer, coxsackievirus and adenovirus receptor, liposome, transduction, extrusion, homogenization, good manufacturing practice, storage stability

## Abstract

Adenovirus (Ad) is a widely studied viral vector for cancer therapy as it can be engineered to cause selective lysis of cancer cells. However, Ad delivery is limited in treating cancers that do not have coxsackievirus and adenovirus receptors (CAR). To overcome this challenge, Ad-encapsulated liposomes were developed that enhance the delivery of Ads and increase therapeutic efficacy. Cationic empty liposomes were manufactured first, to which an anionic Ad were added, which resulted in encapsulated Ad liposomes through charge interaction. Optimization of the liposome formula was carried out with series of formulation variables experiments using an extrusion process, which is ideal for laboratory-scale small batches. Later, the optimized formulation was manufactured with a homogenization technique—A high shear rotor-stator blending, that is ideal for large-scale manufacturing and is in compliance with Good Manufacturing Practices (GMP). Comparative in vitro transduction, physicochemical characterization, long-term storage stability at different temperature conditions, and in vivo animal studies were performed. Ad encapsulated liposomes transduced CAR deficient cells 100-fold more efficiently than the unencapsulated Ad (*p* ≤ 0.0001) in vitro, and 4-fold higher in tumors injected in nude mice in vivo. Both extrusion and homogenization performed similarly–with equivalent in vitro and in vivo transduction efficiencies, physicochemical characterization, and long-term storage stability. Thus, two Ad encapsulated liposomes preparation methods used herein, i.e., extrusion vs. homogenization were equivalent in terms of enhanced Ad performance and long-term storage stability; this will, hopefully, facilitate translation to the clinic.

## 1. Introduction

**Figure 1:**
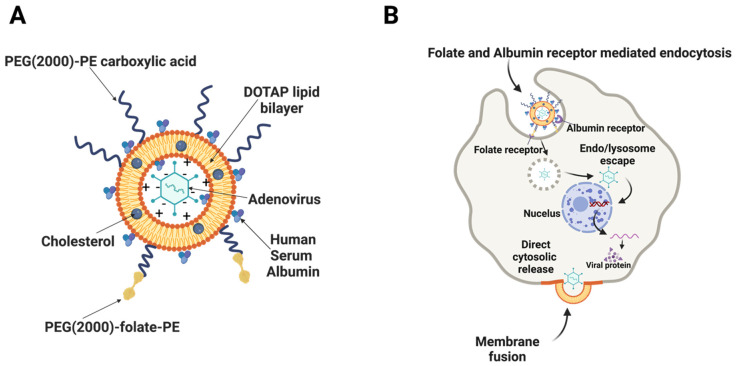
Structure of Ad liposome and its mechanism of action in CAR deficient cancer cells: (**A**) Uni-lamellar or multi-lamellar cationic liposome containing negatively charged Ad. (**B**) Ad liposomes contains folate-conjugated lipid that enhances cellular uptake into folate receptor positive cells by folate receptor-mediated endocytosis. Folate receptors are commonly expressed on numerous types of cancer cells [36,37,38]. Ad liposomes also contains Human Serum Albumin (HSA) and that enhances cellular uptake, possibly via albumin receptor-mediated endocytosis through receptors such as the 60-kDa glycoprotein (gp60) receptor, and Secreted Protein Acidic and Rich in Cysteine (SPARC) [39].

Cancer is a leading cause of death, contributing to nearly 10 million deaths worldwide in 2020 [1]. Cancer arises from genetic alterations that result in disruptions in the highly regulated cell cycle, leading to uncontrolled proliferation and additional mutations. In the process, some cancer cells acquire the ability to evade the immune system by mimicking healthy cells or by releasing immunosuppressive cytokines or chemokines [2]. Therefore, new cancer treatments with novel mechanisms of action and without cross-resistance are required. Oncolytic viruses are primarily immunotherapy agents that selectively replicate in malignant cancer cells, thereby prompting immunogenic cell death. Adenovirus (Ad) are nonenveloped, icosahedral double-stranded DNA viruses that have been developed for transgene delivery in gene therapy applications and as oncolytic anticancer agents [3,4,5,6]. The ability of distinctly designed oncolytic Ad to target tumor cells specifically and to induce systemic anti-cancer immunity with minimal toxicity to non-malignant tissues makes them well-suited for use not only as a primary therapy but also in combination with chemotherapy, targeted pathway inhibition, other immunotherapies, radiation and surgical resection as presurgical neoadjuvant and post-surgical adjuvant therapy [7,8,9,10,11]. Most Ad serotypes need access to coxsackievirus and adenovirus receptors (CAR) to enter and transduce the cancer cells effectively. CAR expression is tremendously heterogenous in cancer types that can limit Ad efficacy in cancer cells with low CAR expression [12,13]. To overcome the need of CAR-dependent cell entry, liposomes are used to encapsulate Ad [14,15]. Liposomes are self-assembled unilamellar or multilamellar vesicles consisting of a lipid bilayer and an aqueous interior compartment, and they have been substantially investigated as carriers of therapeutic agents due to their flexibility in size and biocompatibility [16,17]. Liposomes manufactured by 1,2-dioleoyl-3-trimethylammonium-propane (DOTAP) has demonstrated promising results for the effective gene therapy of DNA, m-RNA and Ad vectors [18,19,20,21]. In the present study, DOTAP liposome formulation was optimized by addition of cholesterol, 1,2-distearoyl-sn-glycero-3-phosphoethanolamine-N-[carboxy(polyethylene glycol)-2000] (PEG(2000)-PE carboxylic acid), 1,2-distearoyl-sn-glycero-3-phosphoethanolamine-N-[folate(polyethylene glycol)-2000] (PEG(2000)-folate-PE), and Human Serum Albumin (HSA). Cationic empty liposomes were manufactured first, to which an anionic Ad were added, which resulted in encapsulated Ad liposomes through charge interaction. These Ad liposomes were able to significantly enhance transduction efficiency of Ad in CAR deficient cancer cells, in vitro via folate receptor and albumin receptor-mediated endocytosis (Figure 1).

Liposome manufacturing by membrane extrusion technique has been studied previously [22,23,24]. Though membrane extrusion is a viable option for a small lab-scale liposome manufacturing, it has several drawbacks in large-scale, good manufacturing practice (GMP) compliant, manufacturing that is essential for clinical studies [25,26]. The pores in the membrane tend to clog, especially when manufacturing concentrated suspensions and/or when the membrane pore sizes are substantially smaller than the liposomes. The clogged membranes cannot be cleared or regenerated and replacing the membranes during aseptic manufacturing can likely compromise the sterility [27,28,29]. Secondly, the membranes are planar disks which must be placed against a flat mechanical support. This design restricts the surface area available for extrusion and may lead to very slow throughput [28]. Lastly, polycarbonate membranes cannot be steam-sterilized in place, with a high degree of quality assurance, because of their inherent fragility [28].

**Figure 2:**
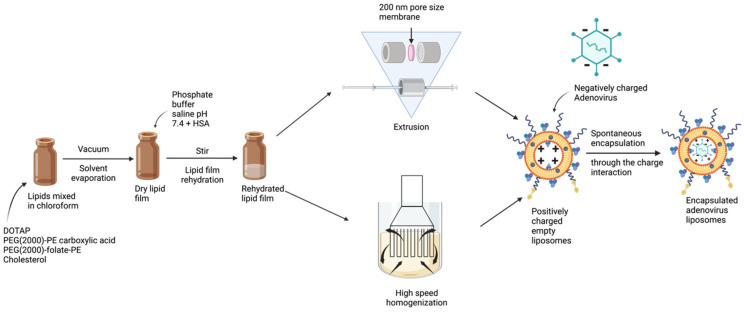
Two processes for manufacturing Ad liposomes: Extrusion (for small-scale) and homogenization (for large scale).

High shear homogenization is a proven liposome manufacturing technique for small to large scale production in a sterile setting with GMP compliance [30,31]. Homogenization speed and mixing time are critical process parameters and need to be optimized [32]. In the present study, development of liposomes was carried out using extrusion and high-speed homogenization techniques (Figure 2). Comparative in vitro transduction, and physicochemical characterization were carried out for Ad liposomes produced by these techniques resulting in identical properties for both manufacturing processes. Long-term storage stability at different storage conditions confirmed that, liposomes manufactured by these techniques equally retained their in vitro transduction efficiency, for one month.

A major challenge related to clinical delivery of viral therapies is the immediate clearance by the liver [33,34]. Although, Ad is administered via intratumoral (IT) injections, adenoviral particles are small enough to extravasate from leaky, tortuous tumor neovessels and enter the systemic circulation [4,35]. Comparative in vivo biodistribution study of Ad liposomes manufactured by two techniques and unencapsulated Ad was carried out. Results demonstrated that Ad liposomes produced by homogenization and extrusion techniques equally reduced liver transduction and increased the tumor transduction. Thus, long-term stable Ad encapsulated liposomes, addressing some of the barriers associated with viral vectors-based therapies, were successfully manufactured by two processes.

## 2. Materials and Methods

### 2.1. Reagents and Cell Lines

Green Fluorescent Protein (GFP) expressing replication-deficient Ad (GFPAd) was purchased from Baylor College of Medicine (Vector: Ad5-CMV-eGFP). Ad-Luciferase (AdLuc) was purchased from Vector BioLabs (Catalog # 1000). 4T1 (mouse breast cancer cells), HEK293 (human embryonic kidney cells) and CT26 (mouse colon cancer cells) cell lines were purchased from American Type Culture Collection (ATCC). A549 (human lung cancer cells), and MCF7 (human breast cancer cells) cell lines were generously provided from the laboratory of Dr. Tony Reid. Dulbecco’s Modified Eagle Medium (DMEM) with high glucose (HyClone Catalog # SH30081.01) was supplemented with 10% of Fetal Bovine Serum (FBS, Corning Catalog # 35-011-CV) and 1% of Pen Strep Glutamine (PSG, Life Technologies Catalog # 10378-016) to prepare the complete media for HEK293, A549, and MCF7 cell culturing. Rosewell Park Memorial Institute (RPMI) 1640 (Gibco Catalog # 11875093) and RPMI 1640 medium no folic acid (Gibco Catalog # 27016021) were supplemented with 10% FBS and 1% of PSG to prepare the complete RPMI (RP-10) for 4T1 and CT26 cell culturing. All cells were cultured at 37 °C and 5% CO_2_ in the complete media.

### 2.2. Manufacturing of Liposomes by Extrusion Technique

DOTAP (Avanti Catalog # 890890C), cholesterol (Sigma-Aldrich Catalog # C3045), PEG(2000)-PE carboxylic acid (Avanti Catalog # 880135P), and PEG(2000)-folate-PE (Avanti Catalog # 880124P) were suspended in chloroform (Sigma Aldrich Catalog # C2432) at molar ratio 1:0.26:0.02:0.01. To make 400 µL of liposome, the amount of each lipid was added as follows: DOTAP 387 nmol, Cholesterol 100 nmol, PEG(2000)-PE carboxylic acid 7.01 nmol and PEG(2000)-folate-PE 3 nmol with 193.13 μL of chloroform in an amber colored vial (Fisher Scientific, Catalog # 03-339-23C). The lipid mixture was vortexed in an amber vial for 30 min at ambient temperature (25 °C). The mixture was vacuumed overnight to form a dry lipid film. The next day, dried lipid film was hydrated with 400 μL of 50 mg mL^−1^ HSA (Sigma-Aldrich catalog#A9511-5G) solution prepared with Phosphate Buffer Saline pH 7.4 (PBS) (Fisher Scientific Catalog # 10010072), while vortexing. The hydrated film was stirred at 600 rpm at 4 °C for 30 min. After 600 rpm stirring at 4 °C for 30 min, the empty liposomes were formed by extruding the lipid solution with Avanti Mini extruder (Avanti Catalog # 610000-1EA) through a 200 nm membrane (Cytiva/Whatman Catalog#10417004), 8 times at room temperature. To these empty liposomes (Df), GFPAd or AdLuc were added, and suspension was incubated for 30 min at ambient room temperature resulting in extruded DOTAP-folate Adenovirus liposomes (Ex Df + Ad). The Ad to DOTAP lipid ratio [Viral Particles (VP): nmol] is 5.17 × 10^7^. Freshly prepared adenovirus liposomes were used for each experiment except for the storage stability studies.

### 2.3. Manufacturing of Liposomes by Homogenization Technique

The dry lipid film preparation and hydration were performed as mentioned in Section 2.2. The batch size was 4 mL, 10× higher than the extruded liposomes. After 600 rpm stirring at 4 °C for 30 min, hydrated films were combined into a 10 mL glass vial. The glass vial was placed in an external water bath maintained at 20 °C and the empty liposomes were formed using a highspeed homogenizer mixer (MXBAOHENG, model RCD-1A) at 18,000 RPM speed for 5 min. To these empty liposomes, GFPAd or AdLuc were added, and suspension was incubated for 30 min at ambient room temperature resulting in homogenization process derived DOTAP-folate Adenovirus liposomes (HMG Df + Ad). The Ad to DOTAP lipid ratio (VP: nmol) is 5.17 × 10^7^. Freshly prepared adenovirus liposomes were used for each experiment except for the storage stability studies.

### 2.4. In Vitro Transduction

Cells were plated overnight at 3 × 10^4^ cells well^−1^ in a 96-well plate at 37 °C and 5% CO_2_ in the complete media. Samples were added to cells (day 1) at a Multiplicity of Infection (MOI) 50 [plaque-forming unit (pfu) per cell] and incubated at 37 °C and 5% CO_2_. GFP fluorescence intensities were measured using a Tecan F PLEX Infinite 200 Pro microplate reader (Tecan Group Ltd., Männedorf, Switzerland) on day 2, 6, 4, 4, and 6 for HEK293, A549, MCF7, 4T1 and CT26, respectively. All the samples were analyzed in triplicates (n = 3). In order to perform background subtraction, intensity values from wells with untreated cells were subtracted from the treated wells.

### 2.5. Liposome Formulation Optimization

Liposome formulation optimization was performed via selection of excipients that have been approved for human use. In this study, different molecular weights of PEG-PE carboxylic acid; PEG(1000)-PE carboxylic acid (Nanosoft Polymers SKU # 2142-1000-100 mg), PEG(5000)-PE carboxylic acid (NANOCS Catalog # PG2-CADS-5k), and PEG(10000)-PE carboxylic acid (NANOCS Catalog # PG2-CADS-10k) were evaluated by in vitro transduction experiments using CAR deficient CT26 cell line. Different molecular weights of PEG-folate-PE; PEG(1000)-folate-PE (Nanosoft Polymers SKU # 4666-1000-50 mg), PEG(3400)-folate-PE (Nanosoft Polymers SKU # 4666-3400-50 mg), and PEG(5000)-folate-PE (Nanosoft Polymers SKU # 4666-5000-50 mg) were evaluated by in vitro transduction experiments using CAR deficient CT26 cell line. Optimization of each excipient was performed by evaluating the effect on transduction of the Ad encapsulated liposomes when each excipient was re-moved from manufacturing. Table 1 outlines formulation compositions of these formulations. In vitro transduction experiments were carried out as per the procedure listed under Section 2.4 and paired *t*-test analysis was performed to calculate *p* values.

### 2.6. Dynamic Light Scattering (DLS) and Zeta Potential Measurements

Mean particle sizes of sample formulations were determined using a Malvern Zetasizer Nano-ZS (Malvern Pananalytical, Malvern, UK). Prior to measurement, samples were diluted with PBS (1:10). For DLS, five acquisitions were taken at 10 s each. The system was used in the auto measuring mode. Mean hydrodynamic size, and Polydispersity Index (PDI) were automatically calculated using the Malvern’s Zetasizer software 8.02 (Malvern Pananalytical, Malvern, UK). For zeta potential, the system was used in the auto measuring mode. Minimum five and maximum fifteen acquisitions were taken with forty-five seconds delay between measurements. Mean zeta potential was automatically calculated using the Malvern’s Zetasizer software. The samples were analyzed in triplicates (n = 3).

### 2.7. Cryo-Transmission Electron Microscopy (Cryo-EM)

Quantifoil carbon R2/2 copper grids (Quantifoil Micro Tools GmbH, Jena, Germany) were glow discharged before sample freezing. The 3 µL of sample solution was applied to the Quantifoil grid and blotting was completed with a Leica EMGP plunger (Leica Microsystems Inc., Deerfield, IL, USA) at room temperature and 95% humidity. Blotting time was set to 3 s without waiting and draining time. The frozen hydrated grid was loaded on a pre-cooled Gatan cryo-transfer holder (Gatan, Inc., Pleasanton, CA, USA) and imaged under a JEOL JEM-2100F transmission microscope (JEOL USA, Peabody, MA, USA), operating at 200 kV. Images were taken at 30,000× magnification, corresponding to 0.27 nm pixel size at specimen space, and recorded on a Gatan OneView CCD (Gatan, Inc., Pleasanton, CA, USA) with SerialEM software (University of Colorado, Boulder, CO, USA) in low-dose mode. Images were processed using Fiji software [40]. Note: Cryo-EM parameters were optimized in order to capture high-resolution images. However, the background noise in micrographs of Ex Df + GFPAd and HMG Df + GFPAd was inevitable due to the high concentration of HSA in them.

### 2.8. Fluorescenece Microscopy

CT26 cells transduced with samples were analyzed under Keyence BZ-X710 microscope (KEYENCE CORPORATION OF AMERICA, IL, USA) with a GFP filter and 470/40 nm excitation wavelength, 525/50 nm emission wavelength and dichroic mirror wavelength 495 nm. Comparative micrographs were captured using 2× and 20× objective lenses.

### 2.9. Long-Term Storage Stability of Ad Liposomes

Empty liposome samples were manufactured by extrusion and homogenization and were placed on stability at 4 °C, −20 °C, and −80 °C. At stability time point, samples were pulled from their storage conditions. To these empty liposomes, GFPAd were added, and suspension was incubated for 30 min at ambient room temperature. Comparative in vitro transduction experiment of aged samples was carried out as per the procedure listed under Section 2.4 along with freshly prepared liposomes manufactured by respective techniques.

### 2.10. In Vivo Biodistribution of Ad Liposomes

All animal experiments were approved by the University of California San Diego—Institutional Animal Care and Use Committee (IACUC). Nu/Nu mice were purchased from the Jackson Laboratory (stock # 002019). Mice were housed in high-efficiency particulate air (HEPA) cages in a specific pathogen-free facility with food and water available ad libitum and a 12 h light/dark cycle. Female mice were used for the experiment, aged between 8 and 12 weeks. The 1 × 10^6^ of CT26 cells in 50 μL PBS were subcutaneously injected to the right flanks of the mice. Tumor formation took approximately 1 week for physical detection. 100 μL of samples containing 1.4 × 10^8^ PFU AdLuc were injected via IT administration. After 5 days, 100 μL (3 mg, ~150 mg/kg) of VivoGlo Luciferin (Promega Catalog # P1041) was injected intraperitoneally into mice 10 min prior to in vivo imaging. Mice were anesthetized with Isoflurane and then imaged in the XENOGEN IVIS 200 Imaging System (XENOGEN Corp., Alameda, CA, USA). Each mouse was placed on its abdomen followed by back and imaged with the same settings: 5 min exposure, medium binning, and F-stop = 1. Average radiance was calculated using Living Image Software 4.0 (Caliper Life Sciences, Waltham, MA, USA) at a threshold of 50%. For tumors, or livers that did not have signal a standard selection area was used, and background radiance was measured at the location of interest.

## 3. Results

### 3.1. Liposome Formulation Optimization

**Figure 3:**
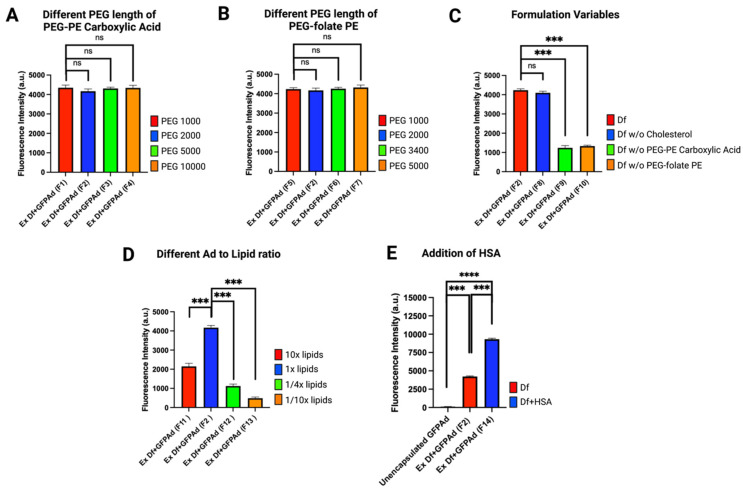
Liposome formulation optimization based on in vitro transduction on CAR deficient CT26 mouse colon cancer cell line at MOI 50 (n = 3): (**A**) Liposomes manufactured with different length of PEG-PE carboxylic acid (*p* value: ns = not significant). (**B**) Liposomes manufactured with different length of PEG-folate-PE (*p* value: ns = not significant). (**C**) Liposomes manufactured by removing one excipient at a time demonstrating significance of PEG-PE carboxylic acid and PEG-folate-PE (*p* value: ns = not significant, *** ≤ 0.001). (**D**) Liposomes manufactured by different Ad to DOTAP lipid ratios (*p* value: *** ≤ 0.001). (**E**) Liposomes manufactured by addition of HSA resulting in 100-fold higher transduction compared to the unencapsulated Ad (*p* value: *** ≤ 0.001, **** ≤ 0.0001).

Fluorescent protein expression is a substitute for viral production and the expression of any protein of interest for gene therapy [41,42]. Liposome formulation optimization was performed using CAR deficient CT26 cell line and infecting at Multiplicity of Infection (MOI) 50. Ex Df + GFPAd Liposomes manufactured using different molecular weights of PEG-PE carboxylic acid; F1 (PEG(1000)-PE carboxylic acid), F2 (PEG(2000)-PE carboxylic acid), F3 (PEG(5000)-PE carboxylic acid) and F4 (PEG(10000)-PE carboxylic acid) were evaluated (Figure 3A). All four liposomes manufactured containing these different molecular weights of PEG-PE carboxylic acid demonstrated similar in vitro transduction efficiency (*p* value > 0.05). In vitro transduction of liposomes manufactured with different molecular weights of PEG-folate-PE; F5 (PEG(1000)-folate-PE), F2 (PEG(2000)-folate-PE), F6 (PEG(3400)-folate-PE), and F7 (PEG(5000)-folate-PE) resulted in equal transduction efficiency (*p* value > 0.05) (Figure 3B). Thus, within these ranges, change in the PEG length for both PEG-PE carboxylic acid and PEG-folate-PE, do not have any impact on the transduction efficiency. Removal of cholesterol from the liposome formula (F8) did not have any significant impact on the in vitro transduction efficiency (*p* value > 0.05) (Figure 3C). This likely means that cholesterol can be completely removed from the liposome formula. However, cholesterol provides fluidity, rigidity and stability for the liposomes therefore addition of cholesterol in liposome formula is considered ideal [17,43]. Removal of PEG(2000)-PE carboxylic acid from the liposome formula (F9), and removal of PEG(2000)-folate-PE (F10) demonstrated significantly lower in vitro transduction efficiency (*p* value = 0.0001, 0.0004, respectively) (Figure 3C). This means that both PEG(2000)-PE carboxylic acid and PEG(2000)-folate-PE are essential excipients that are necessary for high performance of Ad liposomes. In vitro transduction study of various Ad to DOTAP lipid ratios (VP: nmol) in the finished product F11 (5.17 × 10^6^), F2 (5.17 × 10^7^), F12 (2.68 × 10^8^), and F13 (5.17 × 10^8^) demonstrated that the F2 formulation with the ratio 5.17 × 10^7^ is the optimum Ad to DOTAP lipid ratio that provides maximum transduction efficiency on CAR deficient CT26 cell line (Figure 3D). Addition of 50 mg mL^−1^ HSA in the liposome formula (F14) demonstrated ~2.4× higher transduction efficiency compared to the F2 (*p* value = 0.0002) (Figure 3E). In vitro transduction efficiency of F14 was approximately 100-fold higher than the unencapsulated GFPAd (*p* value = 0.0089) for the CAR deficient CT26 cell line.

### 3.2. Comparitve Characterization of Ad Liposomes Produced by Extrusion vs. Homogenization

**Figure 4:**
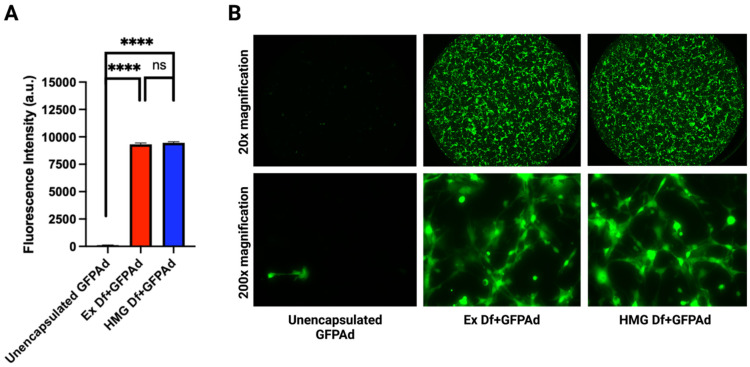
Comparative in vitro transduction of Ad liposomes manufactured by extrusion and homogenization on CAR deficient CT26 cancer cell line (n = 3): (**A**) Liposomes manufactured by extrusion (red bar) and homogenization (blue bar) demonstrated similar transduction (*p* value: ns = not significant). Compared to unencapsulated GFP Ad (yellow bar is not visible in the chart due to a very low fluorescence intensity) both processes demonstrated significantly higher transduction (*p* value: **** ≤ 0.0001) at MOI 50. (**B**) Comparative fluorescence microscopy images of unencapsulated GFPAd, Ex Df + GFPAd, and HMG Df + GFPAd at 20× and 200× magnifications.

**Figure 5:**
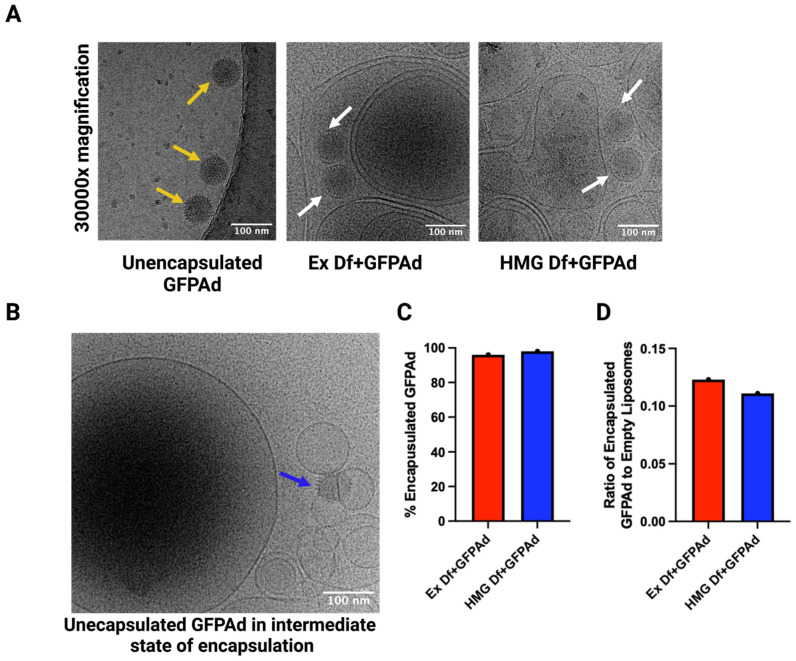
Cryo-EM images of Ad liposomes and unencapsulated Ad: (**A**) Cryo-EM images of unencapsulated GFPAd, Ex Df + GFPAd, and HMG Df + GFPAd at 30000× magnification. Yellow arrows highlight unencapsulated GFPAd without lipid encapsulation and white arrows highlight spherical lipid encapsulation around GFPAd in GFPAd liposomes. (**B**) Cryo-EM image demonstrating intermediate state of virus encapsulation. Blue arrow highlights the unencapsulated GFPAd in intermediate state of encapsulation. All scale bars represent 100 nm. (**C**) % Encapsulation efficiency of GFPAd in Ex Df + GFPAd (red bar = 96%) and HMG Df + GFPAd (blue bar = 98%). (**D**) Ratio of encapsulated GFPAd to empty liposomes in Ex Df + GFPAd (red bar = 0.12) and HMG Df + GFPAd (blue bar = 0.11).

Ad liposomes produced by extrusion (Ex Df + GFPAd), and homogenization (HMG Df + GFPAd) using F14 formulation were tested for comparative in vitro transduction in CAR deficient CT26 cells at MOI 50 (Figure 4A). Ex Df + GFPAd and HMG Df + GFPAd demonstrated similar transduction efficiency (*p* value > 0.05). Compared to the unencapsulated GFPAd, both Ex Df + GFPAd and HMG Df + GFPAd were able to transduce the cells with high efficiency (*p* value < 0.0001). Transduced cells were examined under fluorescence microscope at 20× and 200× magnifications (Figure 4B). It was observed that compared to the unencapsulated GFPAd, both Ex Df + GFPAd and HMG Df + GFPAd were able to transduce high number of CAR deficient CT26 cells. Hydrodynamic size (z-average) and zeta potentials were measured for Ex Df (empty liposomes), Ex Df + GFPAd, HMG Df (empty liposomes), HMG Df + GFPAd, and unencapsulated GFPAd (Table 2). The size distribution by intensity was recorded (Figures S1–S5). The hydrodynamic size (z-average) and zeta potentials were measured for formulations F1–F13 (Table S1). Comparative cryo-EM micrographs were captured (Figure 5A), mechanism of encapsulation was observed and reported (Figure 5B). Cryo-EM micrographs confirmed that the Ad liposomes are both unilamellar and multilamellar vesicles. Encapsulation efficiency and ratio of encapsulated GFPAd to empty liposomes were manually calculated using a set of cryo-EM micrographs. Encapsulation efficiency for Ex Df + GFPAd and HMG Df + GFPAd was found to be 96% and 98%, respectively (Figure 5C). The ratio of encapsulated GFPAd to empty liposomes for Ex Df + GFPAd and HMG Df + GFPAd was found to be 0.12 and 0.11, respectively (Figure 5D). These results indicate that almost all GFPAd are encapsulated using ~10% of the total liposomes.

### 3.3. Comparative In Vitro Transduction of Ad Liposomes Manufactured by Extrusion and Homogenization on CAR Positive and CAR Deficient Cell Lines

**Figure 6:**
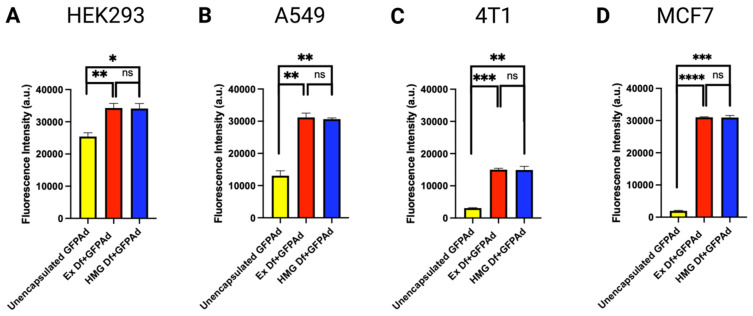
Comparative in vitro transduction of Ad liposomes manufactured by extrusion and homogenization on CAR positive and CAR deficient cell lines at MOI 50 (n = 3): (**A**) In vitro transduction of unencapsulated GFPAd (yellow bar) Ex Df + GFPAd (red bar) and HMG Df + GFPAd (blue bar) on CAR positive HEK293 cells (*p* value: ns = not significant, * ≤ 0.05, ** ≤ 0.01). (**B**) In vitro transduction of unencapsulated GFPAd (yellow bar) Ex Df + GFPAd (red bar) and HMG Df + GFPAd (blue bar) on CAR positive A549 cells (*p* value: ns = not significant, ** ≤ 0.01). (**C**) In vitro transduction of unencapsulated GFPAd (yellow bar) Ex Df + GFPAd (red bar) and HMG Df + GFPAd (blue bar) on CAR deficient 4T1 cells (*p* value: ns = not significant, ** ≤ 0.01, *** ≤ 0.001). (**D**) in vitro transduction of unencapsulated GFPAd (yellow bar) Ex Df + GFPAd (red bar) and HMG Df + GFPAd (blue bar) on CAR deficient MCF7 cells (*p* value: ns = not significant, *** ≤ 0.001, **** ≤ 0.0001).

Comparative in vitro transduction of Ad liposomes manufactured by extrusion and homogenization techniques (F14) was studied on CAR positive transformed but non-cancerous HEK293 human embryonic kidney cells, CAR positive A549 human lung cancer cell line and CAR deficient 4T1 mouse breast cancer cells, and MCF7 human breast cancer cell line at MOI 50 (Figure 6) [44,45,46,47]. Results confirmed that in vitro transduction performance of Ex Df + GFPAd and HMG Df + GFPAd liposomes was found equivalent (*p* value > 0.05) in all cell lines. Transduction efficiency with Df + GFPAd was significantly improved even in CAR positive cell lines at MOI 50.

### 3.4. Long-term Storage Stability of Ad Liposomes

**Figure 7:**
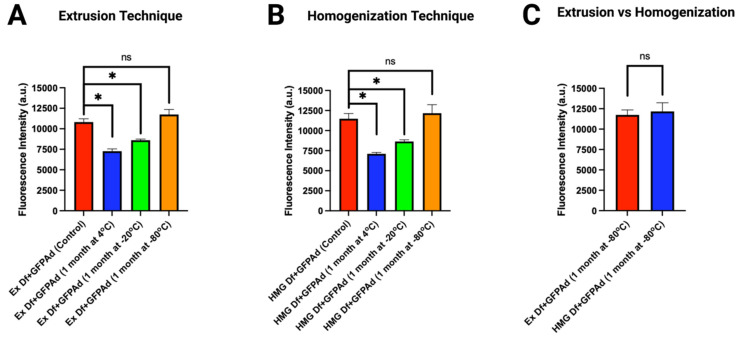
Comparative in vitro transduction of storage stability samples of Ad liposomes manufactured by extrusion and homogenization techniques on CT26 cells at MOI 50 (n = 3): (**A**) In vitro transduction of Ex Df + GFPAd manufactured using 1 month old Ex Df stored at 4 °C (blue bar), −20 °C (green bar), and −80 °C (orange bar), compared with freshly prepared Ex Df + GFPAd (red bar—control sample) (*p* value: ns = not significant, * ≤ 0.05). (**B**) In vitro transduction of HMG Df + GFPAd manufactured using 1 month old HMG Df stored at 4 °C (blue bar), −20 °C (green bar), and −80 °C (orange bar), compared with freshly prepared HMG Df + GFPAd (red bar—control sample) (*p* value: ns = not significant, * ≤ 0.05). (**C**) Comparative in vitro transduction of Ex Df + GFPAd (red bar) and HMG Df + GFPAd (blue bar) at MOI 50–manufactured using 1 month old aged Df stored at −80 °C (*p* value: ns = not significant). Note: Figure 7C is drawn using the data sets taken from (**A**,**B**).

Comparative in vitro transduction of storage stability samples of Ad liposomes manufactured by extrusion and homogenization techniques (F14) was studied on CAR deficient CT26 cell line at MOI 50 (Figure 7). Empty liposomes stored at 4 °C, −20 °C, and −80 °C were pulled after 1 month of storage. To these empty liposomes, GFPAd were added. In vitro transduction of Ad with aged Ex Df (Figure 7A) and HMG Df (Figure 7B) stored at 4 °C and −20 °C was significantly lower compared to the freshly prepared liposomes. However, Ad with aged Df stored at −80 °C retained its transduction efficiency compared to the freshly prepared liposomes (*p* value > 0.05) in a CAR deficient CT26 cell line. Comparative in vitro transduction of Ad with aged Ex Df and HMG Df demonstrated similar transduction efficiency at MOI 50 (*p* value > 0.05) (Figure 7C). The hydrodynamic size (z-average) and zeta potentials were measured for storage stability samples (Table S2).

### 3.5. In Vivo Biodistribution of Ad Liposomes

**Figure 8:**
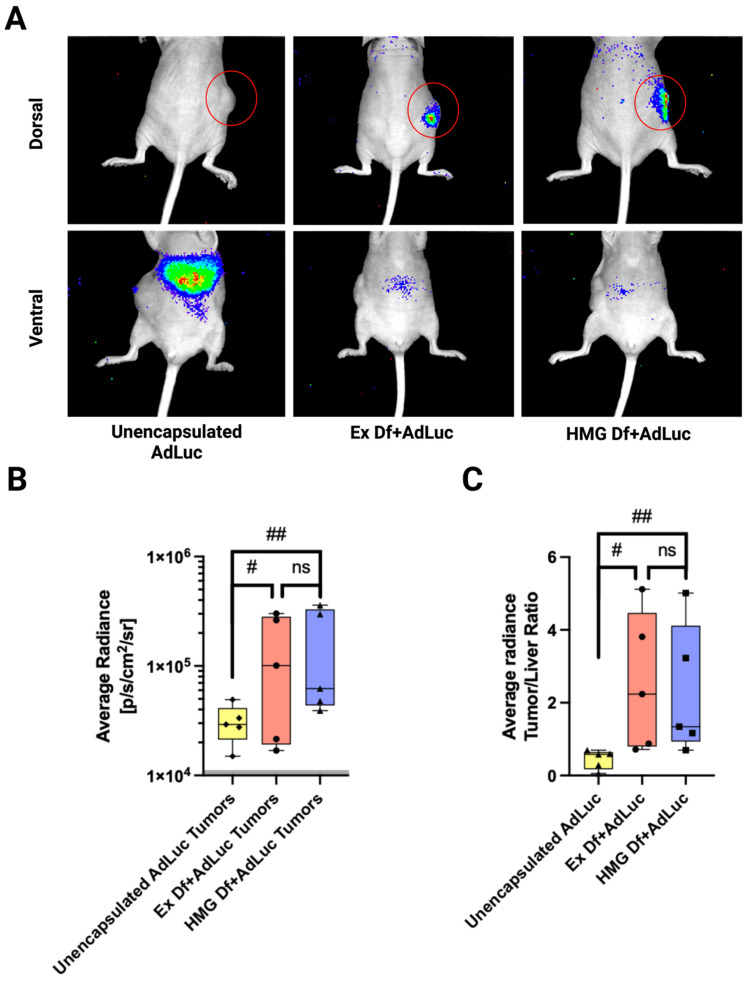
Comparative in vivo biodistribution of Ad liposomes manufactured by extrusion and homogenization techniques: (**A**) The most representative IVIS images of mice IT injected with unencapsulated AdLuc (n = 5), Ex Df + AdLuc (n = 5), and HMG Df + AdLuc (n = 5) at 1.4 × 10^8^ PFU. Both dorsal and ventral sides of each mouse were imaged after 5 days. Red circles highlight injected tumors. (**B**) Average radiance of tumors for Ex Df + AdLuc (n = 5) (red box), HMG Df + AdLuc (n = 5) (blue box), and unencapsulated AdLuc (n = 5) (yellow box) injected mice revealed enhanced transduction of tumors with both Ex Df + AdLuc and HMG Df + AdLuc. Performance of Ex Df + AdLuc, and HMG Df + AdLuc was similar (*p* value: ns = not significant). Radiance < 10,000 p/s/cm^2^/sr (shaded region) is considered background radiance (*p* value: ns = not significant, # = 0.1204 ## = 0.1345). (**C**) For each mouse the ratio of total tumor signal to liver signal demonstrated that Ex Df + AdLuc (red box) and HMG Df + AdLuc (blue box) reduced off-tumor transduction by approximately 4-folds compared to the unencapsulated AdLuc (yellow box) (*p* value: ns = not significant, # = 0.0533 ## = 0.0703).

Comparative in vivo biodistribution was investigated to study transduction efficiency of Ex Df + AdLuc, HMG Df + AdLuc, and unencapsulated AdLuc via IT injections in CAR deficient CT26 tumors implanted in Nu/Nu mice. First, either unencapsulated AdLuc (n = 5) or Ex Df + AdLuc (n = 5) or HMG Df + AdLuc (n = 5) were IT injected with 1.4 × 10^8^ PFU and imaged after 5 days using an IVIS imaging system (Figure 8A). Supporting the in vitro data, both Ex Df + AdLuc and HMG Df + AdLuc enhanced tumor transduction by 4-fold (Figure 8B). The performance of Ex Df + AdLuc, and HMG Df + AdLuc was similar (*p* value > 0.05). The ratio of the total tumor transduction to liver transduction for each group was compared, Ex Df + AdLuc and HMG Df + AdLuc reduced off-tumor transduction by 4-fold (*p* value 0.0533 and 0.0703, respectively) (Figure 8C).

## 4. Discussion

Viral therapy is fundamentally dependent on cell surface proteins such as CAR, thus restricting the ability of vectors to transduce certain types of cancer cells of interest or to overcome tumor heterogeneity. Formulation optimization for encapsulation of adenovirus was successfully performed resulting in enhanced Ad transduction. Enhanced viral delivery is very important for gene delivery applications, direct oncolysis, and immuno-stimulatory molecules induced anti-tumor immunity, that are often restricted by low in vivo therapeutic efficacy [48,49,50,51,52]. In vitro transduction results confirmed that PEG(2000)-PE carboxylic acid, PEG(2000)-folate-PE, and HSA are essential excipients for increased transduction efficiency in CAR deficient cells.

**Figure 9:**
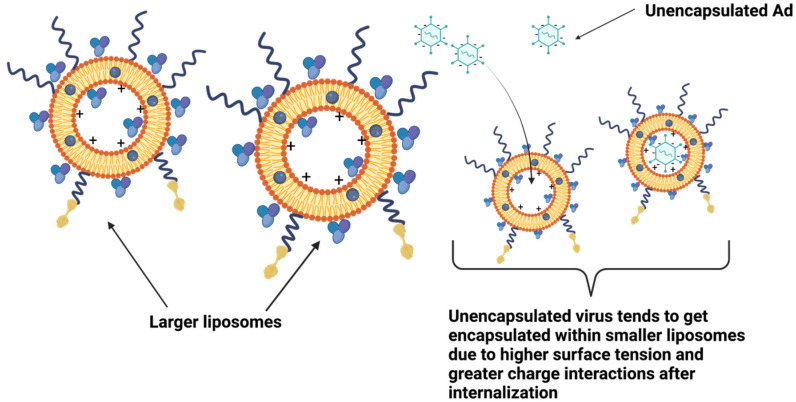
Spontaneous encapsulation of Ad in empty liposomes: unencapsulated virus tends to get encapsulated within smaller liposomes due to higher surface tension and greater charge interactions after internalization.

For Ad liposomes, scalable and GMP complaint manufacturing process is very critical. The main advantage of GMP compliant homogenization technique is the feasibility of manufacturing batches of 1–10 milliliters which is directly scalable to several hundred liters [53,54]. It is hypothesized that the key for efficient and spontaneous virus encapsulation is production of minimum sized liposomes since unencapsulated virus tends to encapsulate within smaller liposomes due to higher surface tension and greater charge interactions after internalization. Both manufacturing processes (extrusion and homogenization) produce minimum sized empty liposomes, thereby driving surface tension induced self-assembly of the virus into the liposome. Post-mixing, unencapsulated virus tends to encapsulate spontaneously within smaller liposomes via charge interactions (Figure 9). Efficient and spontaneous Ad encapsulation is critical for cell membrane fusion and transduction. Small scale extrusion technique and homogenization for larger batch sizes were successfully executed resulting in identical in vitro Ad transduction in various cell lines and similar physio-chemical properties, *viz*., mean hydrodynamic size, zeta potential, and encapsulation efficiency. Long-term storage stability revealed that liposomes manufactured by both processes were able to retain their in vitro transduction efficiency when stored at −80 °C.

For Ad therapies, the liver is a considerable sink for Ad that reach the systemic circulation, which may diminish therapeutic efficacy [33,34]. Oncolytic viruses are commonly injected intratumorally to achieve high levels in the injected tumor(s) while minimizing off-target activity [55,56,57]. With CAR-negative CT26 tumors, intratumoral injection nevertheless resulted in poor transduction of the tumor and primarily led to off-target transduction in the liver. Encapsulated adenovirus in liposomes manufactured by either extrusion or homogenization had much more efficient tumor transduction and minimal off-target activity. Thus, optimized Ad-encapsulated liposome for cancer therapy was successfully manufactured by two processes resulting in enhanced Ad performance and long-term storage stability, providing proof of principle for manufacturing scale up and clinical translation. The present study demonstrates the feasibility for future studies in which we intend to formulate liposomes that encapsulate replicative transgene-armed oncolytic viruses with targeting moieties for the treatment of both liquid and solid tumors.

## Figures and Tables

**Table 1:** Formulation composition for the experiments conducted for optimizing liposome formula.

Formulation ^1^	Lipid Film Composition (Molar Ratio) (DOTAP:Cholesterol: PEG-PE Carboxylic Acid: PEG-Folate-PE)	Ingredient Used/Removed
F1	1:0.26:0.02:0.01	PEG(1000)-PE carboxylic acid
F2	1:0.26:0.02:0.01	PEG(2000)-PE carboxylic acid
F3	1:0.26:0.02:0.01	PEG(5000)-PE carboxylic acid
F4	1:0.26:0.02:0.01	PEG(10000)-PE carboxylic acid
F5	1:0.26:0.02:0.01	PEG(1000)-folate-PE
F6	1:0.26:0.02:0.01	PEG(3400)-folate-PE
F7	1:0.26:0.02:0.01	PEG(5000)-folate-PE
F8	1:0:0.02:0.01	F2-w/o cholesterol
F9	1:0.26:0:0.01	F2-w/o PEG-PE carboxylic acid
F10	1:0.26:0.02:0	F2-w/o PEG-folate PE
F11	1:0.26:0.02:0.01	10× lipid concentration
F12	1:0.26:0.02:0.01	1/4× lipid concentration
F13	1:0.26:0.02:0.01	1/10× lipid concentration
F14	1:0.26:0.02:0.01	F2-with HSA

^1^ In formulations F1–F4; PEG(1000)-PE carboxylic acid, PEG(2000)-PE carboxylic acid, PEG(5000)-PE carboxylic acid, and PEG(10000)-PE carboxylic acid were used, respectively, while using PEG(2000)-folate-PE for all formulations. In formulation F5–F7; PEG(1000)-folate-PE, PEG(3400)-folate-PE, and PEG(5000)-folate-PE were used, respectively, while using PEG(2000)-PE carboxylic acid for all formulations. In formulation F8–F14; PEG(2000)-PE carboxylic acid and PEG(2000)-folate-PE were used. In formulations F11–F13; 10×, 1/4×, and 1/10× lipid amounts were used (compared to the formulation F2) resulting in Ad to DOTAP lipid ratios in the finished product (VP: nmol) 5.17 × 10^6^, 2.68 × 10^8^, and 5.17 × 10^8^, respectively.

**Table 2:** Comparative particle size (z-average) using DLS, and zeta potential of Ad liposomes manufactured using different processes (n = 3).

Formulation	z-Average (nm)	Polydispersity Index (PDI)	Zeta Potential (mV)
Ex Df (Empty Liposomes)	119 ± 5	0.63 ± 0.06	3.26 ± 2.84
Ex Df + GFPAd	140 ± 1	0.53 ± 0.01	−6.05 ± 0.83
HMG Df (Empty Liposomes)	113 ± 1	0.71 ± 0.00	3.80 ± 1.55
HMG Df + GFPAd	135.9 ± 3.6	0.40 ± 0.08	−5.19 ± 1.11
Unencapsulated GFPAd	118 ± 0	0.09 ± 0.00	−2.58 ± 0.31

## Data Availability

The data presented in this study are available in article and supplementary material.

## Supplementary Materials

The following supporting information can be downloaded at: https://www.mdpi.com/article/10.3390/bioengineering9110620/s1. Figures S1–S5: The size distribution by intensity (graphical representation). Table S1: Particle size (z-average) using DLS, and zeta potential of Ad liposomes (F1–F13) manufactured using extrusion process. Table S2: Particle size (z-average) using DLS, and zeta potential of Ad liposomes storage stability samples.
