# Supplementary material for: Development of Adenovirus Containing Liposomes Produced by Extrusion vs. Homogenization: A Comparison for Scale-Up Purposes
Source: Bioengineering (Basel). 2022 Oct 27;9(11):620. doi: 10.3390/bioengineering9110620 (PMC9687354; doi:10.3390/bioengineering9110620)
Supplement: Supplementary file 1 [file bioengineering-09-00620-s001.zip › bioengineering-1967255-supplementary.pdf]

Supplementary Materials

Article

Development of Adenovirus Containing Liposomes Produced by Extrusion vs Homogenization: A Comparison for Scale-up Purposes

Jaimin R. Shah [1,2,3], Tao Dong [1,2,4], Abraham T. Phung [1,2,4], Tony Reid [5], Christopher Larson [5], Ana B. Sanchez [5], Bryan Oronsky [5], Sarah L. Blair [1,6], Omonigho Aisagbonhi [1,7], William C. Trogler [2] and Andrew C. Kummel [2,*]

[1] Moores Cancer Center, University of California San Diego, La Jolla, 92037
[2] Department of Chemistry and Biochemistry, University of California San Diego, La Jolla, 92093
[3] Materials Science and Engineering, University of California San Diego, La Jolla, 92093
[4] Department of NanoEngineering, University of California San Diego, La Jolla, 92093
[5] EpicentRx, Inc., La Jolla, 92037
[6] Department of Surgery, University of California San Diego, La Jolla, 92037
[7] Department of Pathology, University of California San Diego, La Jolla, 92037
* Correspondence: akummel@ucsd.edu

Supplementary Materials

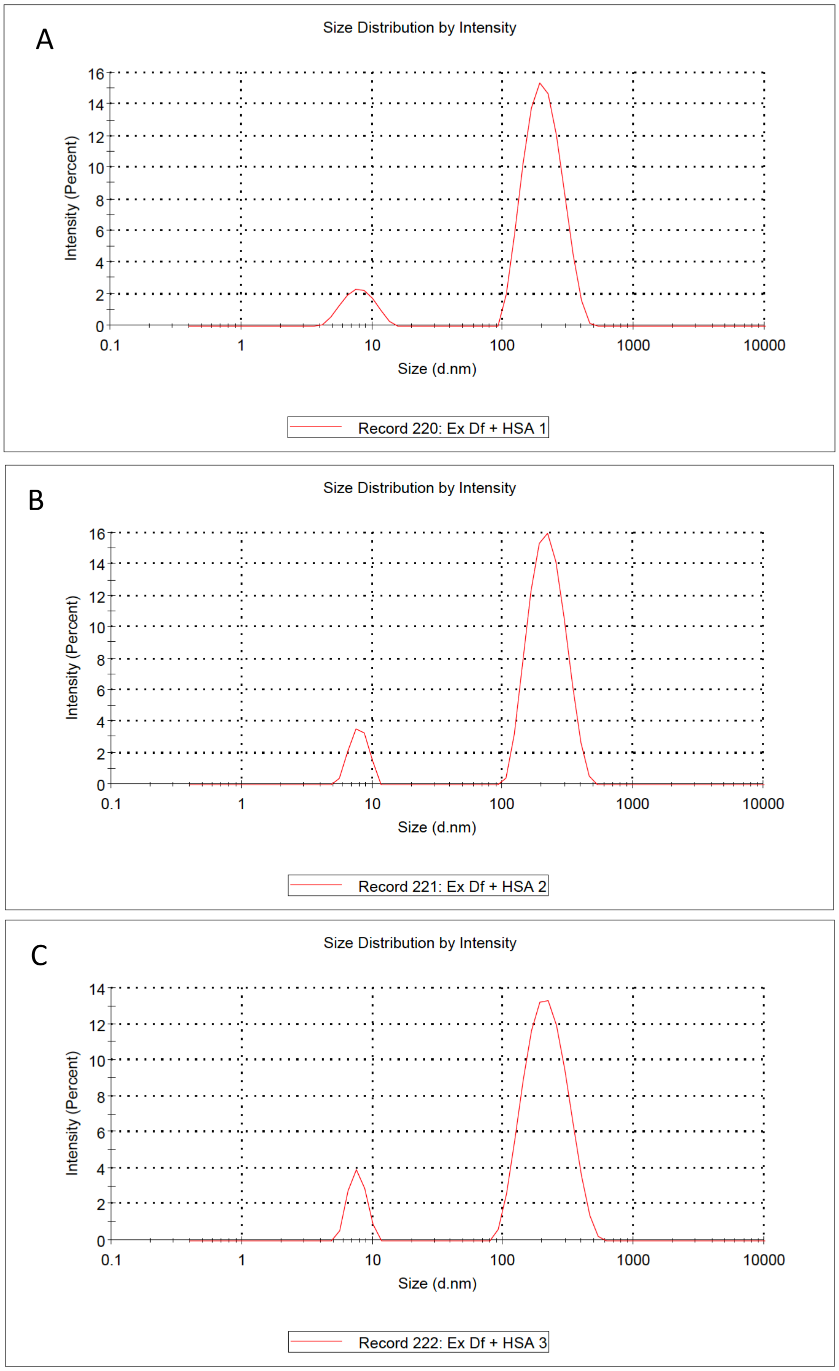

Figure S1: Size distribution by intensity- F14 Ex Df (Empty liposomes) (**A**) Sample 1 Z-Average = 121.0 nm (**B**) Sample 2 Z-Average = 123.8 nm (**C**) Sample 3 Z-Average = 113.5 nm

Supplementary Materials

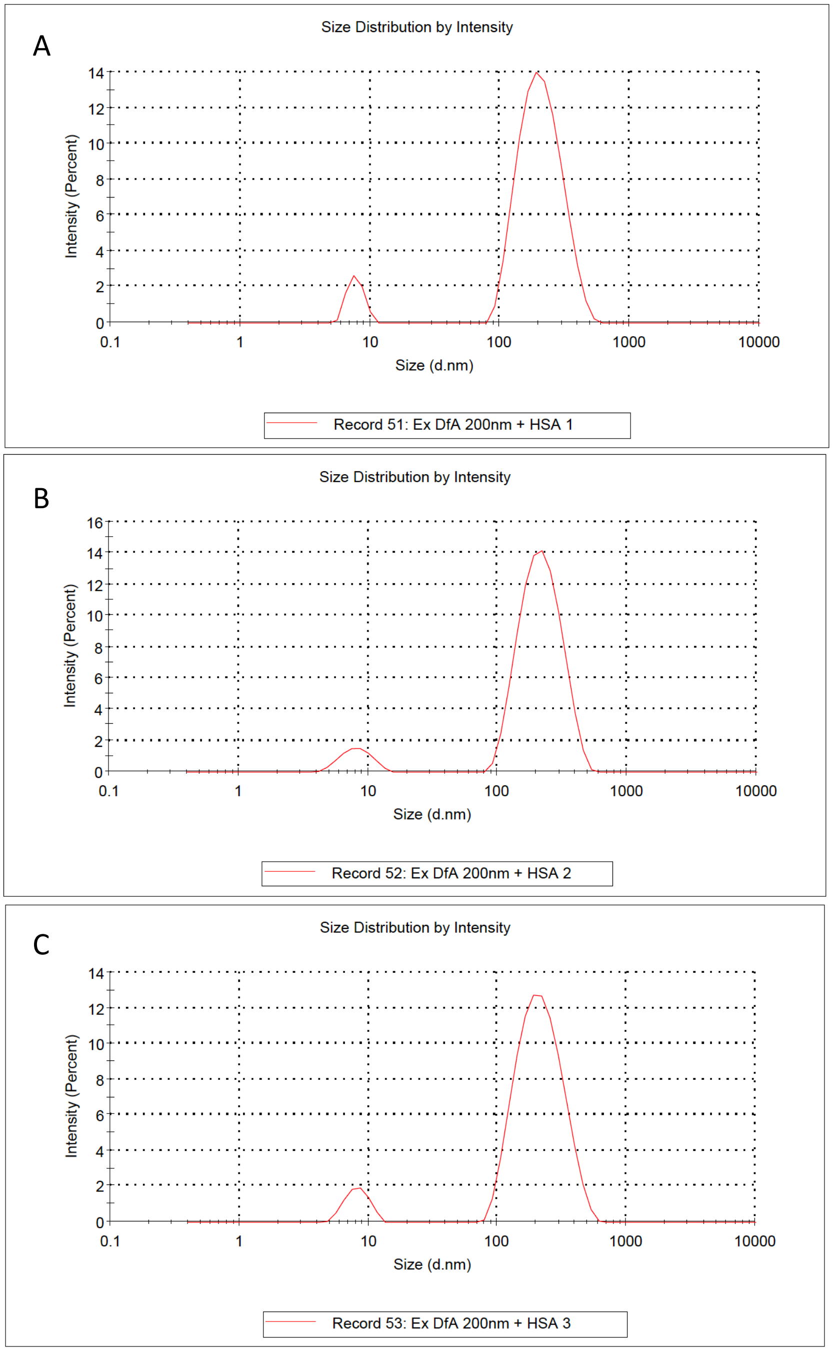

Figure S2: Size distribution by intensity- F14 Ex Df+GFPAd (**A**) Sample 1 Z-Average = 139.2 nm (**B**) Sample 2 Z-Average = 140.8 nm (**C**) Sample 3 Z-Average = 141.5 nm

Supplementary Materials

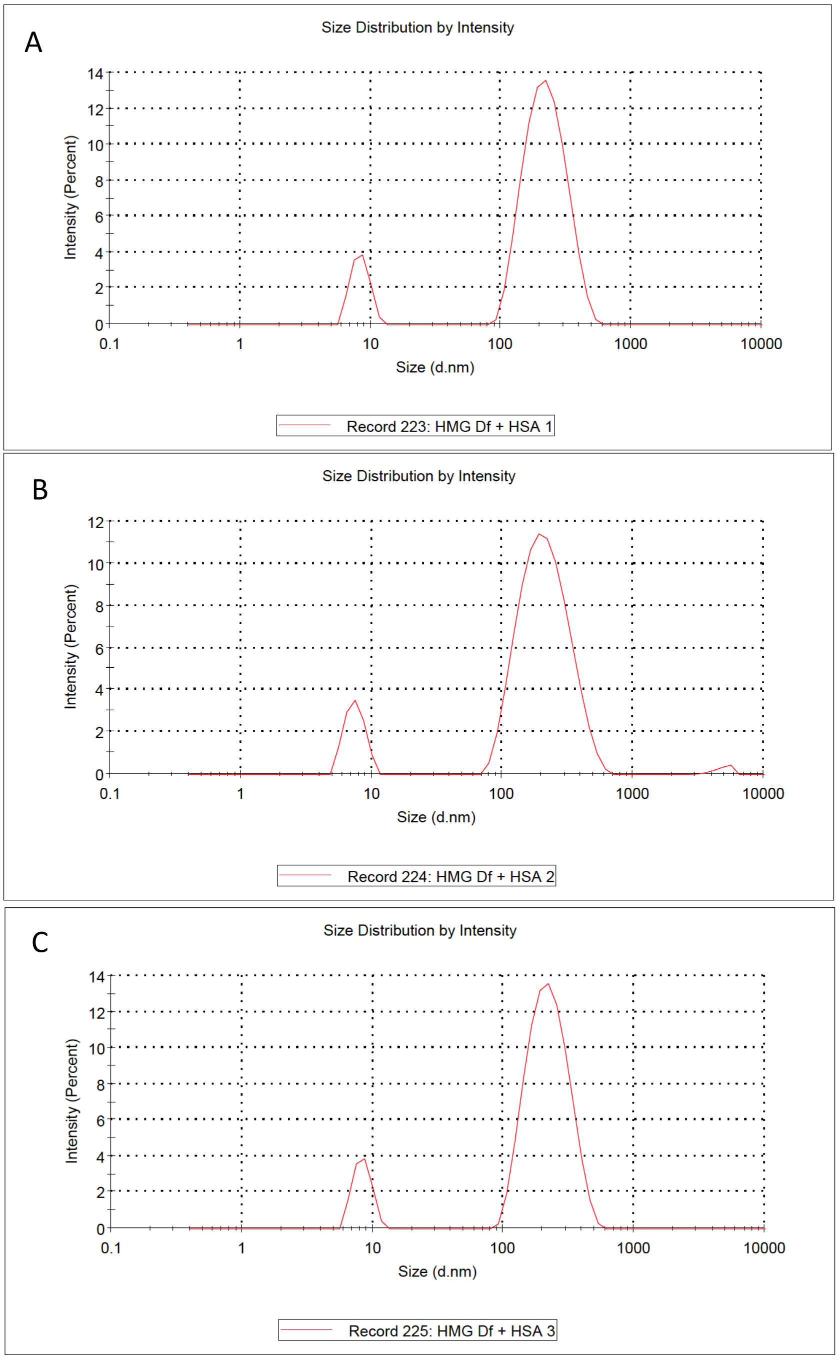

Figure S3: Size distribution by intensity- F14 HMG Df (Empty liposomes) (**A**) Sample 1 Z-Average = 112.4 nm (**B**) Sample 2 Z-Average = 112.3 nm (**C**) Sample 3 Z-Average = 114.2 nm

Supplementary Materials

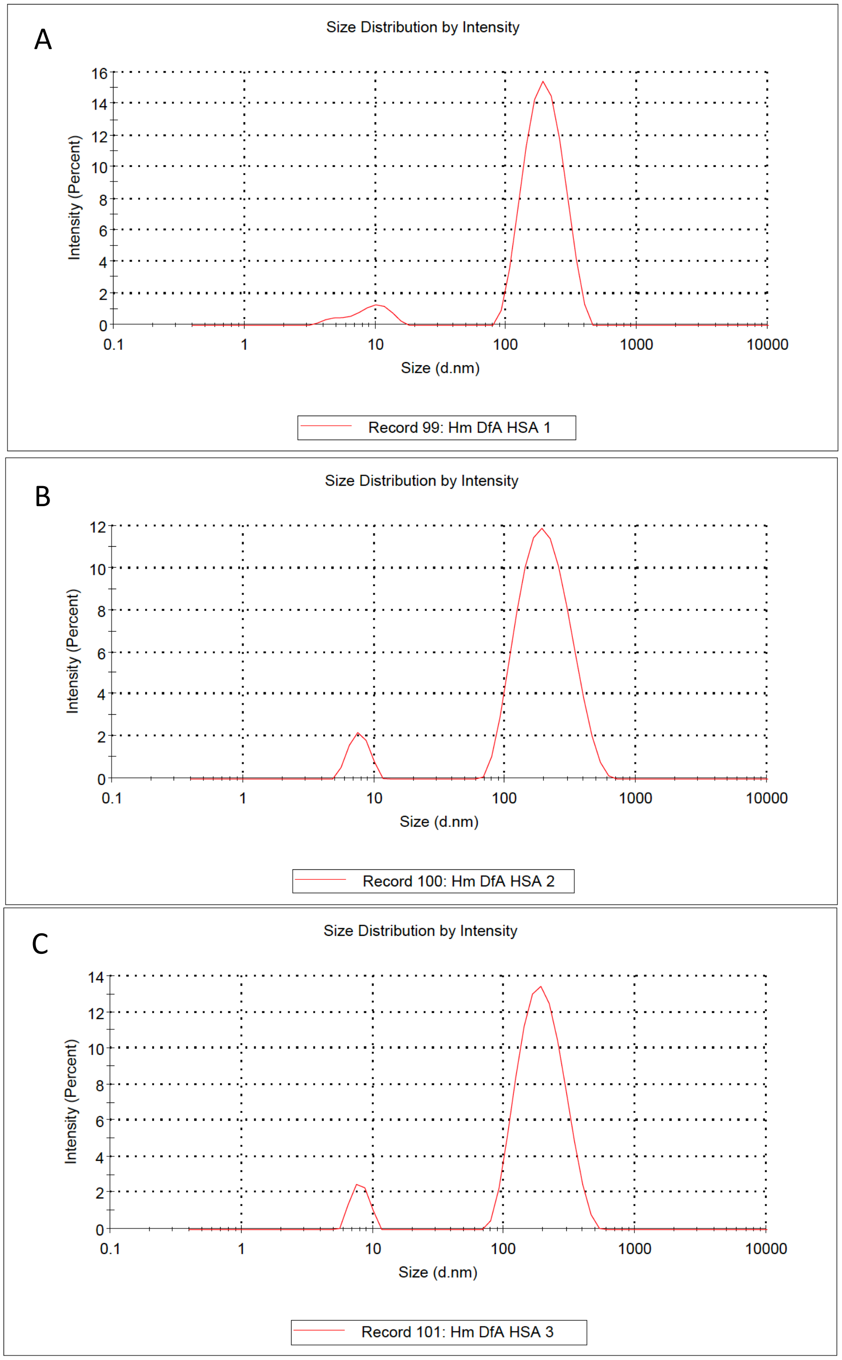

Figure S4: Size distribution by intensity- F14 HMG Df+GFPAd (**A**) Sample 1 Z-Average = 138.2 nm (**B**) Sample 2 Z-Average = 131.8 nm (**C**) Sample 3 Z-Average = 137.9 nm

Supplementary Materials

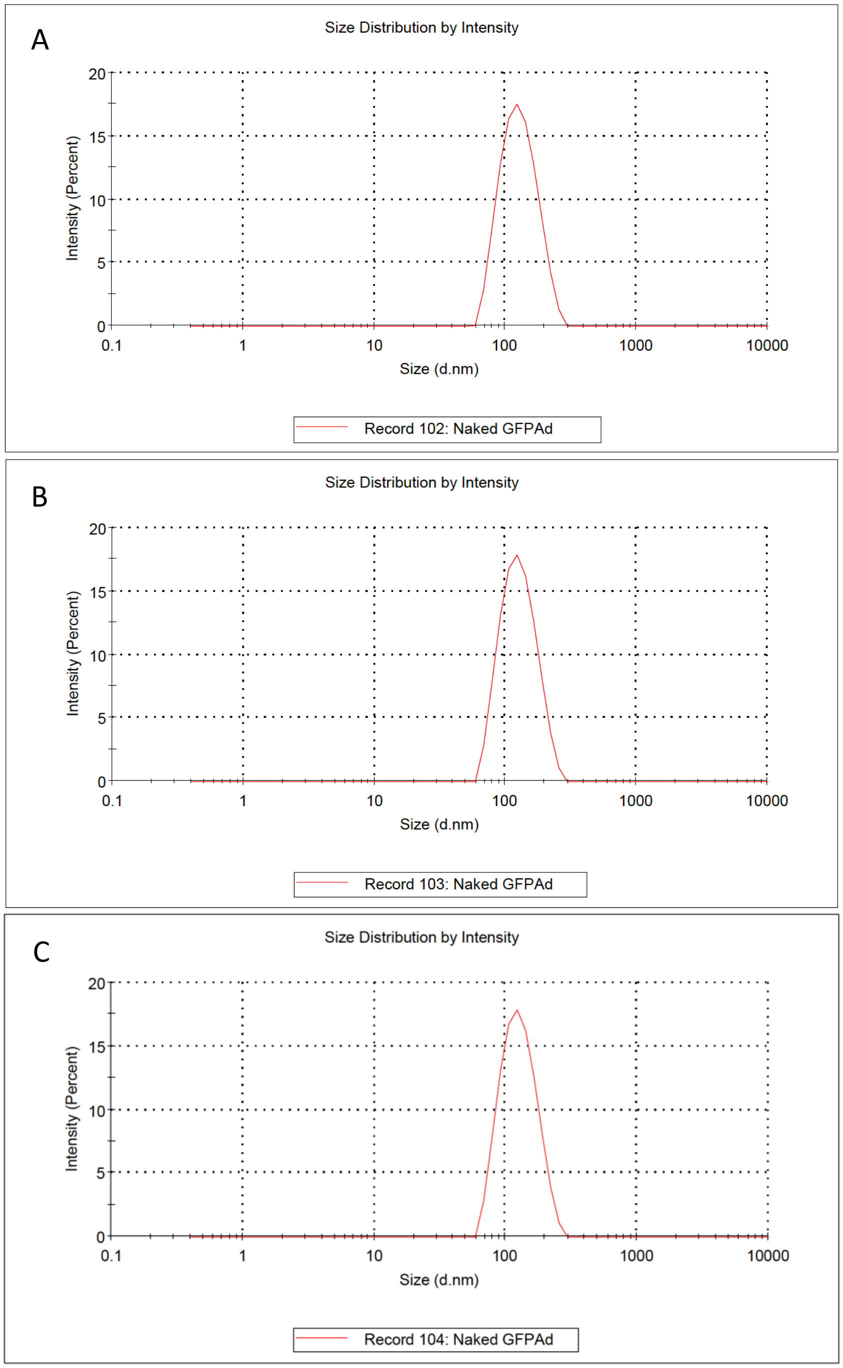

Figure S5: Size distribution by intensity- Unencapsulated GFPAd (**A**) Sample 1 Z-Average = 119.1 nm (**B**) Sample 2 Z-Average = 118.3 nm (**C**) Sample 3 Z-Average = 118.7 nm

Supplementary Materials

Table S1. Particle size (z-average) using DLS, and zeta potential of Ad liposomes manufactured using extrusion process (n = 3).

Formulation[1]	z-average (nm)	Polydispersity Index (PDI)	Zeta Potential (mV)
F1 Empty ExDf	187 ± 2	0.19 ± 0.03	2.93 ± 0.38
F1 ExDf+GFPAd	193 ± 3	0.17 ± 0.03	-3.32 ± 1.54
F2 Empty ExDf	185 ± 5	0.18 ± 0.02	1.52 ± 0.60
F2 ExDf+GFPAd	204 ± 5	0.19 ± 0.05	-1.95 ± 0.48
F3 Empty ExDf	768 ± 78	0.41 ± 0.06	-4.83 ± 0.30
F3 ExDf+GFPAd	829 ± 78	0.40 ± 0.10	-8.54 ± 1.85
F4 Empty ExDf	3069 ± 916	0.17 ± 0.19	12.7 ± 0.4
F4 ExDf+GFPAd	2752 ± 262	0.24 ± 0.23	12.3 ± 0.6
F5 Empty ExDf	302 ± 7	0.44 ± 0.01	2.78 ± 1.76
F5 ExDf+GFPAd	307 ± 20	0.43 ± 0.00	1.92 ± 0.99
F6 Empty ExDf	201 ± 6	0.23 ± 0.04	4.69 ± 0.33
F6 ExDf+GFPAd	208 ± 3	0.26 ± 0.03	0.38 ± 0.06
F7 Empty ExDf	199 ± 4	0.19 ± 0.04	4.09 ± 0.60
F7 ExDf+GFPAd	196 ± 1	0.20 ± 0.01	0.30 ± 0.12
F8 Empty ExDf	194 ± 6	0.27 ± 0.03	-1.14 ± 0.94
F8 ExDf+GFPAd	223 ± 8	0.25 ± 0.03	-0.66 ± 0.99
F9 Empty ExDf	1223 ± 115	0.18 ± 0.16	33.8 ± 1.6
F9 ExDf+GFPAd	2887 ± 172	1.00 ± 0.00	36.0 ± 2.3
F10 Empty ExDf	249 ± 3	0.36 ± 0.03	4.55 ± 1.01
F10 ExDf+GFPAd	313 ± 10	0.22 ± 0.13	3.88 ± 0.47
F11 Empty ExDf	184 ± 1	0.10 ± 0.04	7.21 ± 0.69
F11 ExDf+GFPAd	179 ± 1	0.13 ± 0.02	3.47 ± 0.44
F12 Empty ExDf	173 ± 1	0.21 ± 0.02	-6.43 ± 2.24
F12 ExDf+GFPAd	177 ± 3	0.23 ± 0.03	-8.23 ± 2.21
F13 Empty ExDf	162 ± 3	0.19 ± 0.04	-3.82 ± 1.53
F13 ExDf+GFPAd	159 ± 4	0.17 ± 0.07	-7.84 ± 0.53

[1]In formulations F1 – F4; PEG(1000)-PE carboxylic acid, PEG(2000)-PE carboxylic acid, PEG(5000)-PE carboxylic acid, and PEG(10000)-PE carboxylic acid were used respectively while using PEG(2000)-folate-PE for all formulations. In formulation F5 – F7; PEG(1000)-folate-PE, PEG(3400)-folate-PE, and PEG(5000)-folate-PE were used respectively while using PEG(2000)-PE carboxylic acid for all formulations. In formulation F8 – F13; PEG(2000)-PE carboxylic acid and PEG(2000)-folate-PE were used. In formulations F11 – F13; 10x, 1/4x, and 1/10x lipid amounts were used (compared to the formulation F2) resulting in Ad to DOTAP lipid ratios in the finished product (VP : nmol) 5.17×10^6, 2.68×10^8, and 5.17×10^8 respectively.

Supplementary Materials

Table S2. Particle size (z-average) using DLS, and zeta potential of F14 Ad liposomes storage stability (1 month) samples manufactured using extrusion and homogenization processes (n = 3).

Formulation	z-average (nm)	Polydispersity Index (PDI)	Zeta Potential (mV)
4°C ExDf	121 ± 6	0.72 ± 0.08	2.60 ± 0.31
4°C ExDf +GFPAd	122 ± 9	0.69 ± 0.11	-6.03 ± 1.14
-20°C ExDf	318 ± 5	0.52 ± 0.09	1.43 ± 0.66
-20°C ExDf +GFPAd	338 ± 17	0.56 ± 0.08	-5.09 ± 1.35
-80°C ExDf	521 ± 37	0.36 ± 0.03	2.20 ± 0.43
-80°C ExDf +GFPAd	509 ± 24	0.27 ± 0.09	-5.28 ± 0.65
4°C HMGDf	131 ± 6	0.62 ± 0.04	3.07 ± 1.37
4°C HMGDf +GFPAd	122 ± 4	0.73 ± 0.07	-4.43 ± 0.09
-20°C HMGDf	319 ± 6	0.49 ± 0.04	2.91 ± 0.19
-20°C HMGDf +GFPAd	330 ± 24	0.47 ± 0.04	-3.58 ± 0.48
-80°C HMGDf	494 ± 74	0.37 ± 0.03	2.56 ± 0.70
-80°C HMGDf +GFPAd	504 ± 35	0.34 ± 0.06	-3.49 ± 0.49
